# Non-HDL cholesterol and long-term follow-up outcomes in patients with metabolic syndrome

**DOI:** 10.1186/s12944-023-01923-y

**Published:** 2023-10-04

**Authors:** Fatemeh Vazirian, Susan Darroudi, Hamid Reza Rahimi, MohamadReza Latifi, Behrouz Shakeri, Samaneh Abolbashari, Amir Hooshang Mohammadpour, Habibollah Esmaily, Mohsen Mouhebati, Sara Samadi, Majid Ghayour Mobarhan

**Affiliations:** 1 School of Pharmacy, Mashhad University of Medical Sciences, Mashhad, Iran; 2 International UNESCO center for Health-Related Basic Sciences and Human Nutrition, Mashhad University of Medical Sciences, Mashhad, Iran; 3 Human Research Center, Mashhad University of Medical Sciences, Mashhad, Iran; 4 Department of Biology, Mashhad Branch, Islamic Azad University, Mashhad, Iran; 5 Antimicrobial Resistance Research Center, Mashhad University of Medical Sciences, Mashhad, Iran; 6 Department of Clinical Pharmacy, School of Pharmacy, Mashhad University of Medical Sciences, Mashhad, Iran; 7 Department of Biostatistics, School of Health, Mashhad University of Medical Sciences, Mashhad, Iran; 8 Social Determinants of Health Research Center, Mashhad University of Medical Sciences, Mashhad, Iran; 9 Cardiovascular Research Center, School of Medicine, Mashhad University of Medical Sciences, Mashhad, Iran; 10 Department of Internal Medicine, Faculty of Medicine, Mashhad University of Medical Sciences, Mashhad, Iran; 11 Metabolic Syndrome Research Center, Mashhad University of Medical Sciences, Mashhad, Iran

**Keywords:** non-HDL-C, Metabolic syndrome, Cohort study, Waist circumference, Triglyceride, Blood pressure, HDL

## Abstract

**Background:**

Non-high-density lipoprotein-cholesterol (non-HDL-C) has been identified as a potential biomarker for metabolic syndrome (MetS). However, its predictive capability for MetS varies among different ethnic groups, necessitating further investigation. This study aimed to assess the role of non-HDL-C in the early diagnosis of MetS in the Iranian population through a longitudinal study with a 10-year follow-up period.

**Methods:**

Our study enrolled 4684 individuals from the MASHAD (Mashhad Stroke and Heart Atherosclerotic Disorder) cohort who were followed for 10 years to examine the association between non-HDL-C and the incidence of MetS. Additionally, the contribution of individual MetS components to the overall burden was evaluated.

**Results:**

A total of 1599 subjects developed MetS, while 3085 did not. Non-HDL-C levels ≥ 130 were associated with a 42% higher risk of developing MetS (relative risk (RR), 1.42; 95% confidence interval (CI), 1.25–1.62). Regarding MetS components, elevated waist circumference (WC) showed the strongest association with MetS incidence (RR, 2.32; 95% CI, 1.45–2.9), whereas triglyceride (TG) levels ≥ 150 mg/dL demonstrated the weakest association (RR, 1.23; 95% CI, 1.04–1.46). Additionally, higher HDL-C levels were reported to be 20% protective against the risk of MetS (RR, 0.8; 95% CI, 0.73–0.86). Moreover, fasting blood glucose (FBG) levels ≥ 100 mg/dL were not significantly linked to MetS burden, while systolic blood pressure (BP) levels ≥ 130 mmHg or diastolic BP levels ≥ 85 mmHg increased the risk of MetS incidence (RR, 1.25; 95% CI: 1.11–1.41).

**Conclusions:**

Elevated non-HDL-C and increased WC serve as significant predictors of MetS in Iranians. Strategies targeting non-HDL-C levels and weight loss should be emphasized to mitigate the risk of MetS development.

## Background

Metabolic syndrome (MetS) is defined as a group of cardiovascular risk factors, including glucose disorders accompanied by dyslipidemia, which significantly increase the risk of cardiovascular disease (CVD) events and the prevalence of type 2 diabetes mellitus [1–3]. Given the rising global prevalence of MetS, epidemiological studies are required to investigate its prevalence and associated risk factors across diverse groups of people [4]. In recent studies, two definitions of MetS have been utilized and compared [5]. The modified National Cholesterol Education Program Adult Treatment Panel III (ATP III) characterizes MetS in meeting at least three of five criteria, which include blood pressure ≥ 130/85 mmHg, waist circumference (WC) over 102 cm (men) or 88 cm (women), fasting triglyceride (TG) level ≥ 150 mg/dL, fasting high-density lipoprotein-cholesterol (HDL-C) < 40 mg/dL (men) or 50 mg/dL (women), and fasting blood glucose (FBG) ≥ 100 mg/dL [6]. On the other hand, the International Diabetes Foundation (IDF) criteria for defining MetS involve the presence of obesity based on specific cutoff points for each ethnicity, in addition to meeting at least two of the ATP III criteria [4]. That is, IDF characterizes MetS more specifically, while ATP III diagnoses MetS with higher sensitivity [1]. Emerging evidence indicates that individuals with MetS are at 50–60% higher risk of cardiovascular events; therefore, early diagnosis of MetS can potentially decrease the rate of mortality and morbidity [7].

Multiple studies have highlighted the potential role of atherogenic dyslipidemia in the development of MetS, and non-high-density lipoprotein cholesterol (non-HDL-C) has been proposed as an appropriate marker for identifying MetS prevalence, as it reflects all atherogenic particles [8–11]. Unlike LDL-C, non-HDL-C refers to the cholesterol content found in all the lipoproteins that contribute to atherosclerosis. Therefore, subtracting HDL cholesterol from the total cholesterol yields the non-HDL cholesterol value, which represents the cholesterol carried by all the lipoproteins except HDL. Regarding the linkage between MetS and non-HDL-C, previous evidence demonstrated that individuals with MetS had higher levels of non-HDL-C [12]. Non-HDL-C offers the advantage of not being affected by the prandial situation and is easily applicable in clinical practice [13]. In addition, the predictive ability of non-HDL-C may vary in different ethnicities, emphasizing the need to validate the association between MetS and non-HDL-C among diverse populations [14]. We aimed to investigate the explicit and long-term contribution of non-HDL-C to the incidence of MetS among an Iranian population, utilizing recent data and a 10-year follow-up period.

## Methods

### Study design

The Mashhad stroke and heart atherosclerotic disorder (MASHAD) study commenced in 2010 and is scheduled to extend until 2020 [15]. The population size of Mashhad, obtained from the national Iranian census conducted in 2006, was used to estimate the total population. Participants for the study were selected from three specific regions in Mashhad, situated in the northeastern part of Iran, employing a stratified cluster random sampling method. The MASHAD cohort study recruited subjects aged 35–65 years who had no previous history of coronary artery disease (CAD), stroke, or peripheral arterial disease [15]. Additionally, subjects were not taking any medications for hypertension, diabetes, or abnormal lipid levels. Individuals who completed follow-up were evaluated based on a cardiovascular disease questionnaire and electrocardiography. The eligibility of the individuals was determined through physical examination and medical interviews conducted by cardiologists. The medical examinations were performed by two interventional cardiologists and one electrophysiologist using data obtained from computerized tomography (CT) angiography, angiography, stress echocardiography, and exercise tolerance testing. Prior to participation, all the study participants provided informed written consent. The study protocol was approved by the Ethics Committee of Mashhad University of Medical Sciences.

### Follow-up

**Fig. 1:**
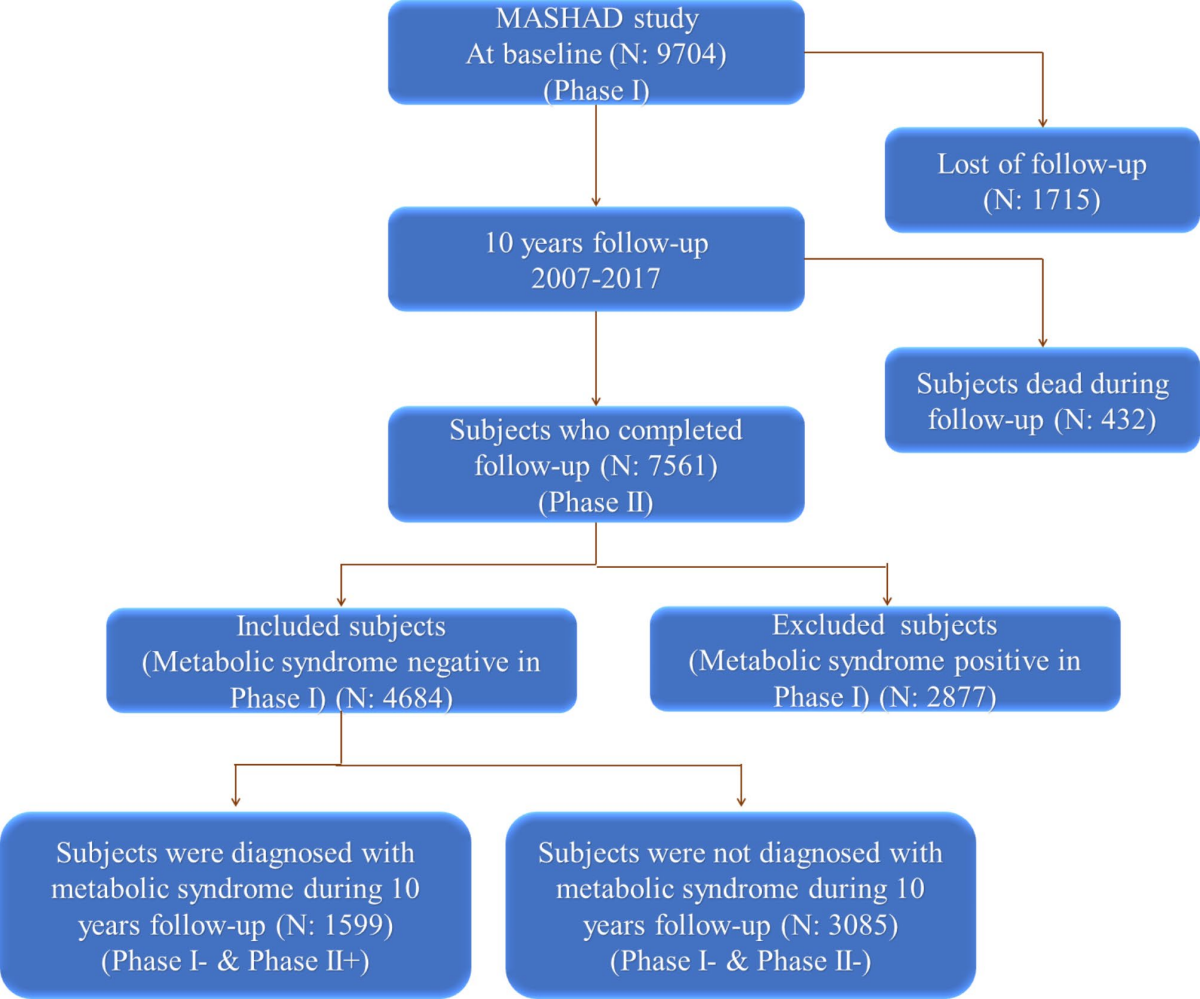
Flowchart of the study

Over the 10-year follow-up period, individuals were examined for MetS burden. The incidence of MetS was considered our primary end point and was adjudicated by two independent cardiologists who were blinded to all the participants` results. The diagnosis of MetS in the present study was based on the criteria set by IDF with specific cutoff points for the Middle East population [16]. The criteria for defining abdominal obesity using WC vary across different ethnic groups and global regions. Although IDF provides specific cutoff points for Europeans (94 cm in men and 80 cm in women), there is a lack of updated data on the normal range of WC in Middle Eastern regions. Consequently, the IDF recommends using the European cutoff points as a reference to define the normal range of WC in these regions [17, 18]. According to these criteria, subjects with central obesity based on WC ≥ 94 cm in males and ≥ 80 cm in females plus having at least two of the following factors were considered positive for MetS: (1): systolic blood pressure (SBP) ≥ 130 mmHg or diastolic blood pressure (DBP) ≥ 85 mmHg, or currently taking hypertension medication; (2) HDL < 40 mg/dL in males and < 50 mg/dL in females, or currently taking medication; (3) TG ≥ 150 mg/dL, or currently taking medication for higher TG levels; and (4) FBG ≥ 100 mg/dL, or previously diagnosed with type 2 diabetes. Based on these criteria, a total of 1599 subjects were diagnosed with MetS (Fig. 1).

### Demographic, anthropometric, and biochemical data

The data on demographic and anthropometric variables, including age, sex, weight, BMI, WC, hip circumference (HC), waist hip ratio (WHR), SBP, and DBP, were gathered. The levels of blood glucose, cholesterol, TG, HDL-C, low-density lipoprotein-cholesterol (LDL-C), non-HDL-C, high-sensitivity C-reactive protein (hsCRP), and uric acid were measured [19]. To measure total cholesterol, LDL-C, TG, and HDL-C, enzyme-linked assays on a multiple sample analyzer (Parsazmun, Karaj, Iran) were used [19]. To calculate the non–HDL-C level, the HDL concentration was subtracted from the total cholesterol level. The standard suggested cutoff point for non-HDL-C (130 mg/dL) was applied in our study.

### Statistical analyses

All statistical analyses were performed using SPSS software, version 22. Mean ± SD, median and interquartile range were reported for normally and nonnormally distributed parameters. Baseline characteristics of participants were compared by Student’s t test for normally distributed data and χ2 for categorical data. To analyze parameters with a skewed distribution, the Mann–Whitney test was used. Demographic and biochemical variables were compared between the two groups of participants. The association between non-HDL-C and MetS and its components was evaluated using Cox regression model analysis after adjustment for age, sex, marriage status, job status and education level. Finally, *P* values less than 0.05 were considered significant.

## Results

### Baseline characteristics of participants

A total of 4684 participants were investigated and were divided into two groups: healthy individuals and MetS patients. Accordingly, 1599 individuals (31% men) with a mean age of 47.03 years old were evaluated in the MetS group, while the healthy controls comprised 3085 subjects (52.6% men) with an average age of 46.15 years old. All anthropometric and biochemical values differed significantly between the two groups except for HDL-C level and smoking (Table 1).

**Table 1:** Baseline characteristics of patients according to have MetS after 10-year follow-up in MASHAD cohort study

Variables		Healthy		MetS	*P* value
N (4684)		3085		1599	
***Anthropometrics***					
Age (year)		46.15 ± 8.01		47.03 ± 7.81	< 0.001
Sex (n%)	Male (n%)	1622 (52.6%)		496 (31%)	< 0.001
	Female (n%)	1463 (47.4%)		1103 (69%)	
Smoking	No	1170 (73.2%)		2137 (69.3%)	0.061
	Ex smoker	130 (8.1%)		285 (9.2%)	
	Current	299 (18.7%)		663 (21.5%)	
Marriage	Single	13 (0.8%)		17 (0.6%)	< 0.001
	Married	1482 (92.7%)		2967 (96.2%)	
	Divorced	28 (1.8%)		31 (1%)	
	Widow	76 (4.8%)		70 (2.3%)	
Job	Employee	539 (33.7%)		1511 (49%)	< 0.001
	Unemployed	933 (58.3%)		1277 (41.4%)	
	Retired	127 (7.9%)	295 (9.6%)		
Education level	Low	902 (56.4%)	1384 (44.9%)		< 0.001
	Moderate	550 (34.4%)	1223 (39.7%)		
	High	147 (9.2%)	475 (15.4%)		
Weight (kg)		66.67 ± 11.36		73.03 ± 11.73	< 0.001
BMI (kg/m^2^)		25.45 ± 4.17		28.7 ± 4.06	< 0.001
WC (cm)		88.84 ± 10.78		96.21 ± 11.46	< 0.001
HC (cm)		99.8 ± 8.39		105.08 ± 8.52	< 0.001
WHR (cm)		0.55 ± 0.07		0.6 ± 0.07	< 0.001
***Blood glucose***					
Glucose (mg/dL)		82.38 ± 25.6		85.81 ± 27.79	< 0.001
***Lipid profile***					
Cholesterol (mg/dL)		183.98 ± 36.02		190.87 ± 37.59	< 0.001
TG (mg/dL)		92(69–124)		111(86–137)	< 0.001
HDL-C (mg/dL)		45.09 ± 10.39		44.57 ± 10.02	0.1
LDL-C (mg/dL)		117.65 ± 33.95		146.29 ± 34.65	0.003
Non-HDL-C (mg/dL)		114.57 ± 32.7		138.88 ± 34.14	< 0.001
***Blood pressure***					
SBP (mmHg)		114.71 ± 14.95		117.46 ± 14.32	< 0.001
DBP (mmHg)		75.48 ± 10.72		76.67 ± 9.51	< 0.001
***Inflammation***					
HsCRP (mg/dL)		1.3(0.82–2.4)		1.69(1.02–3.47)	< 0.001
Uric acid (mg/dL)		4.44 ± 1.24		4.58 ± 1.57	0.001

Data are reported as mean ± SD except for TG and hsCRP, that were reported as median (interquartile range).

Abbreviations: N, number; BMI, body mass index; WC, waist circumference; HC, hip circumference; WHR, waist hip ratio; SBP, systolic blood pressure; DBP, diastolic blood pressure; TG, triglyceride; HDL-C, high-density lipoprotein-cholesterol; LDL-C, low-density lipoprotein-cholesterol; non-HDL-C, non-high-density lipoprotein-cholesterol; HsCRP, high-sensitivity C-reactive protein.

### The prevalence of the components of MetS among the subjects

**Fig. 2:**
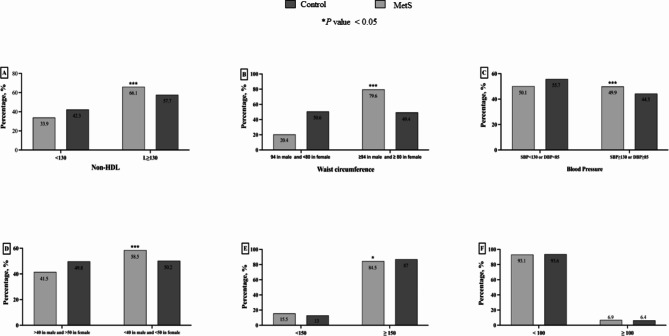
The prevalence of MetS criteria among the two groups of controls and subjects with MetS. The prevalence of MetS is plotted against (A) non-HDL-C (B) waist circumference (C) blood pressure (D) HDL-C (E) triglyceride, and (F) fasting blood sugar. *P value < 0.05. Abbreviations: non-HDL-C, non-high-density lipoprotein cholesterol; HDL-C, high-density lipoprotein cholesterol; FBG, fasting blood glucose

As illustrated in Fig. 2, the prevalence of each criterion for MetS was compared in the two groups of healthy individuals and MetS patients. All the criteria differed significantly between the two groups except for FBG ≥ 100 mg/dL.

In comparison to the control group, a total of 66.1% of individuals who were diagnosed with MetS after follow-up had non-HDL-C ≥ 130 mg/dL (*P* < 0.001). Furthermore, approximately 79.6% of subjects in the MetS group comprised men with WC ≥ 94 cm and women who had WC ≥ 80 cm compared to the control group (p < 0.001). The results showed that 49.9% of the group with MetS and 44.3% of the subjects in the healthy group had SBP ≥ 130 mmHg or DBP ≥ 85 mmHg (p < 0.001). A total of 58.5% of individuals with MetS included men with HDL < 40 mg/dL and women with HDL < 50 mg/dL in comparison to the control group (p < 0.001). There was no significant difference regarding FBG among the two groups of participants (p = 0.53).

### Association of non-HDL-C and five components of MetS with the occurrence of MetS after a 10-year follow-up

As shown in Table 2, non-HDL-C was stratified by levels, and non-HDL-C < 130 mg/dL was considered as a reference in our analysis. The relative risk (RR) for elevated non-HDL-C was estimated as 1.42 (95% confidence interval (CI), 1.25–1.62) for the occurrence of MetS after 10 years. Regarding the five components of MetS, elevated WC had the highest contribution to the incidence of MetS among patients (RR, 2.32; 95% CI, 1.45–2.9), and TG levels ≥ 150 mg/dL indicated the lowest association with the occurrence of MetS (RR, 1.23; 95% CI, 1.04–1.46). Our analysis demonstrated that the association between MetS incidence and FBG ≥ 100 mg/dL was not statistically significant after a 10-year follow-up (RR, 1.08; 95% CI, 0.84–1.37). Moreover, there was a significant contribution of higher HDL-C levels to the occurrence of MetS (RR, 0.8; 95% CI, 0.73–0.86). We also found that BP ≥ 130 mmHg or DBP ≥ 85 mmHg could increase the risk of MetS (RR, 1.25; 95% CI, 1.11–1.41).

**Table 2:** Risk of metabolic syndrome after 10 years follow-up according to MetS components

	*P* value	RR	95% CI for Exp(B)	
			Lower	Upper
Non-HDL-C ≥130 (< 130, ref)	0.000	1. 428	1. 259	1. 62
WC ≥ 94 in male and ≥ 80 in female	0.000	2.32	1.459	2.9
BP SBP ≥ 130 or DBP ≥ 85	0.003	1.255	1.112	1.416
HDL-C < 40 in male and < 50 in female	0.039	0.801	0.738	0.869
TG ≥ 150	0.000	1.236	1.041	1.468
FBG ≥ 100	0.11	1.08	0.848	1.375

Abbreviations: MetS, metabolic syndrome; RR, risk ratio; CI, confidence interval; non-HDL-C, non-high-density lipoprotein-cholesterol; WC, waist circumference; BP, blood pressure; HDL-C, high-density lipoprotein-cholesterol; TG, triglyceride; FBG, fasting blood glucose.

Note. The risk ratio was adjusted for age, sex, marriage status, job status and education level.

## Discussion

The key finding of the present study is that elevated non-HDL-C levels are significantly associated with MetS burden. Individuals with increased non-HDL-C levels are at approximately 42% greater risk of clustering metabolic abnormalities, which can further promote the likelihood of CVD development in their lifetime.

A series of metabolic disorders, including elevated TG, blood sugar, obesity, and hypertension, are essential for MetS development. These characteristics of MetS contribute to multiple pathogenic mechanisms, such as inflammation, oxidative stress, and endothelial dysfunction, which in turn promote atherosclerosis [20, 21]. Non-HDL-C reflects all the atherogenic lipid fractions and is an associated factor for atherosclerosis. Given the common cross-linking pathophysiology pathways shared with MetS and plaque formation, an increased level of non-HDL-C is expected to have a predictive ability in determining individuals susceptible to MetS burden [22].

Consistent with our results, a study conducted in 2022 and 60,799 individuals reported that both non-HDL-C levels higher than 247 mg/dL and 118–247 mg/dL were significantly associated with an increased risk of MetS (odds ratio (OR),17.18; 95% CI, 14.29–20.65 and OR, 3.08; 95% CI, 2.77–3.42, respectively) [23]. In addition, in a recent meta-analysis investigating studies from 2000 to 2021 among 17,860 subjects, a significant linkage between non-HDL-C and MetS was reported. The analysis demonstrated that non-HDL-C was a robust predictor of MetS in adults (OR, 3.53; 95% CI, 2.29–4.78; n, 8,549) and in children (OR, 2.27; 95% CI, 1.65–2.90; n, 9,311). Moreover, when considering the two different definitions for MetS, non-HDL-C still showed a significant contribution to the development of MetS based on ATP III (OR, 3.77; 95% CI, 2.14–5.39; n, 12,490) and IDF (OR, 2.71; 95% CI, 1.98–3.44; n, 5,370). Since non-HDL-C was associated with the risk of MetS utilizing both MetS definitions, measurement of non-HDL-C could predict the subjects at elevated risk of MetS regardless of MetS criteria. This meta-analysis also reported that non-HDL-C had a notable linkage with MetS-associated factors, including hypertriglyceridemia, obesity, diabetes, and hypertension [24].

In contrast, Lee kh. et al. investigated 511 women to find the relationship between non-HDL-C and MetS using ATP III and IDF criteria. As demonstrated by this study, by comparing non-HDL-C > 151 mg/dL to non-HDL-C < 122 mg/dL, non-HDL-C was a predictive marker for MetS according to the ATP III definition (OR, 4.005; 95% CI, 1.151–13.939), but the association between non-HDL-C and MetS using IDF criteria was not statistically significant (OR, 1.77; 95% CI, 0.51–6.16) [25]. Looking at the inconsistent findings between the study by Lee kh. et al., and the present study, cross-sectional studies cannot provide causality and long-term effects of non-HDL-C with respect to MetS incidence. In addition, focusing only on the female population may not provide a comprehensive representation of the total society. Further investigations in large population studies involving both male and female participants are required to better understand the association between non-HDL-C and the development of MetS.

Our findings indicated that among the five MetS components, WC had the highest contribution to the MetS burden after a 10-year follow-up. As indicated by our results, elevated WC was responsible for an approximately 2.3-fold higher risk of metabolic disorders after adjustment for age, sex, marriage status, job status and education level. In agreement with our findings, a recent study conducted on 5,026 individuals showed higher WC as a significant marker in determining subjects having greater odds of MetS incidence in both men and women (men: OR, 6.38; 95% CI, 5.07–8.02 and women: OR, 3.98; 95% CI, 3.39–4.69) [26]. The observed increase in both non-HDL-C and WC among the participants may be influenced by dietary intake and behavioral lifestyle, which have a role in abnormal metabolic status and further contribute to a higher risk of MetS burden. A sedentary lifestyle and lack of physical activity, consuming a high-calorie diet and unhealthy fat and sugars lead to both lipid abnormalities and weight gain that can further increase the likelihood of having elevated WC and non-HDL-C levels [27, 28]. Moreover, some other reasons can contribute to higher WC and non-HDL-C, such as hormonal imbalance, including thyroid dysfunction and insulin resistance, which highlight the importance of educating subjects about regular monitoring and health check-ups [29, 30]. Reductions in non-HDL-C and WC will subsequently reduce the risk of MetS-associated risk factors and MetS burden through different pathways, including reducing atherogenic lipid factors, modulating inflammatory and oxidative stress pathways, and enhancing endothelial function [31, 32].

The present study demonstrated that individuals with elevated BP and TG are exposed to an increased risk of having abnormal metabolic disorders in long-term follow-up. Consistent with our evidence, a recent cohort study recruiting 2,935 middle-aged Chinese individuals with a 3-year follow-up showed a higher risk of MetS incidence in participants with higher BP than in the normotensive group (hazard ratio (HR), 1.823; 95% CI, 1.538–2.162) [33]. With respect to the significant association between TGs and metabolic disorders in our study, investigating the population in southern Iran demonstrated a notable linkage between high TGs and MetS burden [34].

Evidence obtained from our study demonstrated a long-term association of HDL with abnormal metabolic disorders. We found a statistically significant association between reduced HDL-C levels and the risk of MetS among the Iranian population by considering HDL < 40 mg/dL in men and HDL < 50 mg/dL in women as reference levels (RR, 0.8; 95% CI, 0.73–0.86). Our finding was consistent with previous evidence that displayed a reverse relationship between HDL-C concentration and MetS [3]. In contrast, this association was found to be insignificant by some prospective investigations that compared two groups of healthy individuals and cardiovascular events [35, 36]. Furthermore, multiple studies have demonstrated various antiatherogenic properties of HDL including antioxidant effects, HDL lipid peroxidation, and cholesterol efflux in patients with metabolic disorders [37–39].

With respect to the association between FBG and MetS incidence, our results demonstrated no linkage between FBG and metabolic disorders after a 10-year follow-up. FBG ≥ 100 mg/dL was not significantly associated with the risk of MetS (RR, 1.08; 95% CI, 0.85–1.38). Consistent with our finding, the same insignificant contribution of FBG to MetS development was found in a study recruiting the Iranian participants [40].

The present study recruited a large population and evaluated the association of non-HDL-C and other MetS components with the risk of metabolic abnormalities over a 10-year follow-up. Our results identified non-HDL-C, an easily applicable clinical measurement, as a robust marker for the prediction of MetS among the Iranian population.

Based on the definition criteria by IDF, the national prevalence of MetS is approximately 43.5% (42.7–44.4) in the Iranian population [41]. Several previous studies showed the predictive value of non-HDL-C and diet in the likelihood of MetS prevalence among the Iranian population [22, 42, 43].

### Study strengths and limitations

The current study is among the first to explore the association between non-HDL-C and specific MetS components, focusing on a large Iranian population and demonstrating the linkage of those components with MetS prevalence in a longitudinal design. Our study addressed the research gap regarding the association of non-HDL-C with the incidence of MetS and its components over a long follow-up period. Our findings have clinical implications and provide information for health care settings involved in MetS management and prevention by indicating the predictive value of each MetS component and the long-term impact of higher non-HDL-C levels on the development of MetS. Some limitations in our study should be addressed. As we focused on a specific population (Iranian subjects), the generalizability of our findings is limited. Different races may have variations in genetic background, lifestyle, and health care factors, which may influence the risk and prevalence of MetS. Moreover, although we studied the long-term effects of non-HDL-C and different MetS components on the prevalence of MetS over a 10-year follow-up, further clinical trials and prospective cohorts could establish causality for the associations. Furthermore, we adopted the IDF criteria for MetS diagnosis in the present study, which may limit the comparability of our findings to other studies using different criteria for MetS definition. Finally, no measurement of apolipoprotein-B (APO-B) concentration was provided in the present study, which may provide additional insights into the linkage between MetS incidence and lipid profile. Future research can further expand upon our findings and consider the external validity of our results through inclusion of different ethnicities, implementation of interventional studies, and measurement of additional biomarkers such as APO-B to enhance the accuracy of prediction in MetS burden.

## Conclusion

An explicit association of non-HDL-C with abnormal metabolic disorders was found in our study in which individuals with elevated non-HDL-C were exposed to an approximately 42% increased risk of MetS in their lifetime. The present study suggested that in terms of establishing a platform for the prevention of MetS prevalence, clinicians should consider non-HDL-C lowering therapy among subjects with high non-HDL-C concentrations. Furthermore, among the various components of MetS, WC was found to have the highest contribution to metabolic disorders, as higher WC was associated with a 2.3 times greater risk of MetS incidence after a 10-year follow-up, demonstrating that weight loss strategies, specifically abdominal fat management, can benefit subjects susceptible to MetS development.

## Data Availability

Datasets analyzed during the study are available from the corresponding author upon reasonable request.

## List of abbreviations

MetS: Metabolic syndrome
CVD: Cardiovascular disease
ATP III: Adult Treatment Panel III
WC: Waist circumference
TG: Triglyceride
HDL: High-density lipoprotein cholesterol
FBG: Fasting blood glucose
IDF: International Diabetes Foundation
Non-HDL-C: Non-high-density lipoprotein cholesterol
MASHAD: Mashhad stroke and heart atherosclerotic disorder
CAD: Coronary artery disease
CT: Computerized tomography
HTN: Hypertension
BMI: Body mass index
HC: Hip circumference
WHR: Waist hip ratio
SBP: Systolic blood pressure
DBP: Diastolic blood pressure
LDL: Low-density lipoprotein cholesterol
HsCRP: High-sensitivity C-reactive protein
RR: Relative risk
CI: Confidence interval
OR: Odds ratio
HR: Hazard ratio
APO-B: Apolipoprotein B
